# Intra-articular osteoid osteoma of tempromandibular joint: A case report

**DOI:** 10.1016/j.ijscr.2019.07.070

**Published:** 2019-08-01

**Authors:** Sabah Abdulaziz Issa, Hussein Ali Abdulnabi, Ahmed Salih Hussien Alshewered

**Affiliations:** a Department of Oral and Maxillofacial Surgery, Medical City Complex, Bab Al Muadham, 10047, Baghdad, Iraq; b Clinical Oncology, Baghdad Radiotherapy and Nuclear Medicine Center, Medical City Complex, Bab Al Muadham, 10047, Baghdad, Iraq

**Keywords:** NSAIDs, non-steroidal anti-inflammatory drugs, TMJ, temporo-mandibular joint, CT, computed tomography, Osteoid osteoma, Temporo-mandibular joint, Craniofacial neoplasm, Osteoblastoma, Case report

## Abstract

**Introduction:**

An osteoid osteoma is a benign bone neoplasm with limited growth potential, characterized by significant nocturnal pain that usually responds to non-steroidal anti-inflammatory drugs (NSAIDs). The tumor may occur in any part of the skeleton, most commonly in the lower extremities and vertebrae.

**Presentation of case:**

A 46-year-old female was diagnosed with a rare case of an intra-articular variant of osteoid osteoma, involving the articular eminence and glenoid fossa of the temporo-mandibular joint (TMJ).

**Discussion:**

The tumor presented as a painful progressive swelling in the right pre-auricular area that had lasted for more than 2 years and which had been previously treated as a TMJ disorder. Computed tomography revealed a well-defined heterogeneous nidus involving the articular eminence of the TMJ. Surgical excision was performed and histological examination confirmed the diagnosis. In this article, in addition to describing the case, all cases of osteoid osteoma of the jaws and TMJ reported in the English-language literature are reviewed.

**Conclusion:**

Osteoid osteoma involving the TMJ is a rare presentation and may exhibit ambiguous symptoms, which could lead to misdiagnosis and delayed treatment.

## Highlights

• Osteoid osteoma is a benign bone neoplasm, with significant nocturnal pain that responds to NSAIDs.
• A case of an intra-articular osteoid osteoma involving the articular eminence and glenoid fossa of TMJ in a 46-years-old female is reported.
• CT scan imaging is helpful in displaying the typical radiographic features and localization of the lesion.

## 1 Introduction

Osteoid osteoma is a benign, solitary neoplasm of bone, first characterized in 1935 by Jaffe [1]. It usually involves the long bones and is characterized by significant nocturnal pain that usually responds to non-steroidal anti-inflammatory drugs (NSAIDs) [2]. This tumor comprises about 10% of all benign bone tumors and occurs most frequently during the second decade of life, although its occurrence in a wider age-range has been documented; it is three-times more common in males than females [3]. Osteoid osteoma occurs as a nidus of active remodeling bone within a vascular stroma, surrounded by a reactive sclerotic cortex, and has limited growth potential, reaching a maximum size of just 1.5–2 cm [4,5]. Depending on its clinical presentation, treatment ranges from conservative measures, such as treatment with NSAIDs in the acute stage, to more invasive procedures, for example surgical excision, which can bring immediate pain-relief [5]. The long bones of the lower extremities and the vertebrae are the bones most commonly affected by this tumor, with the proximal femur being the most common site [4,6]. However, osteoid osteoma may occur in any part of the skeleton, including joints, showing an intra-articular variant of the lesion [7]. Few cases have been reported to occur in the craniofacial complex; of those reported that have affected the jaws, a marked predilection for the mandible was seen [5,6].

Here, we present a case of osteoid osteoma of the temporo-mandibular joint (TMJ) involving the articular eminence, which, to our knowledge, has been rarely reported in the literature [5,8].

This work has been reported in line with the SCARE criteria [40].

## 2 Case report

A 46-year-old female presented to the morning consultation clinic of the Oral and Maxillofacial Surgery Department at our institute, complaining of progressive swelling and pain in her right pre-auricular area which had persisted for more than 2 years. The patient reported a history of traumatic incidents with a hard object in the same area, several months prior to the onset of symptoms. The intensity of pain had started to progressively increase, although it was partially responding to NSAIDs. The patient had undergone a TMJ washing procedure and had received an occlusal splint, but this had not alleviated the complaint.

**Fig. 1:**
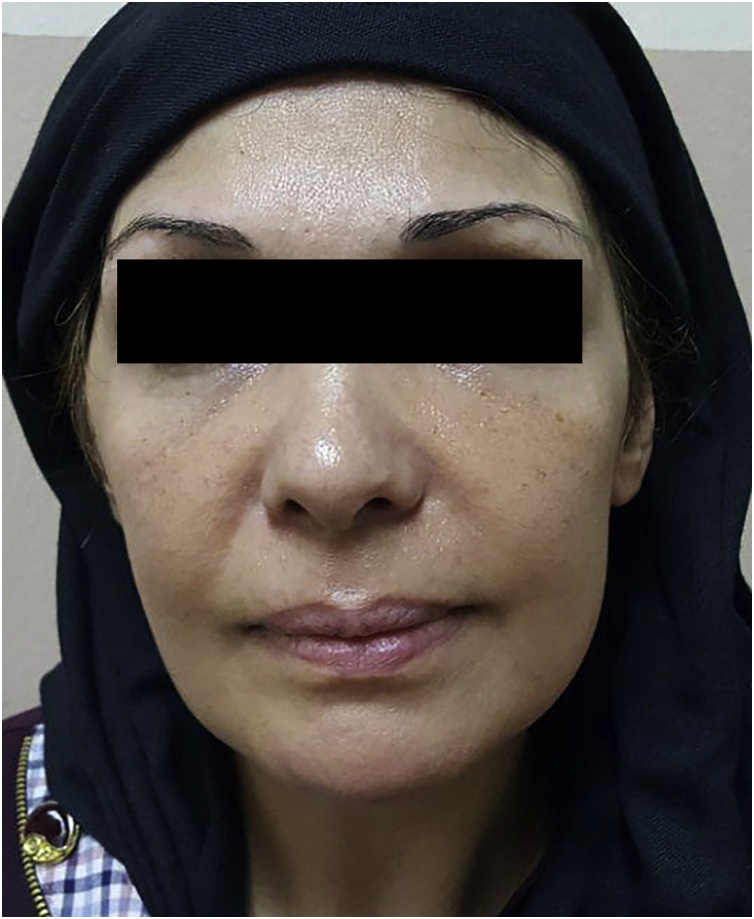
Right-sided Pre-auricular swelling.

**Fig. 2:**
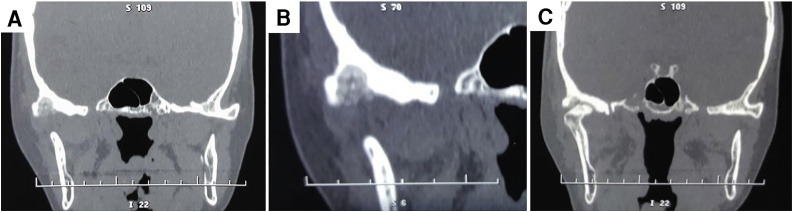
Coronal CT slice demonstrates a well-defined osteolytic lesion in the skull base (A). (B) Focused image shows a heterogeneous nidus contains multiple hyperdense masses with diffused sclerosis of the adjacent bone. (C) The lesion extends posteriorly into the glenoid fossa of the TMJ.

A physical examination revealed a 4 × 2.5 cm swelling with a faded border in the patient’s right TMJ region. The overlying skin was normal in color (Fig. 1). The swelling was firm and very tender to palpation. The patient’s ability to open her mouth was adequate, albeit with a slight deviation of the mandible toward the right-side. There was no clicking or other sound from the TMJ during examination. A computed tomography (CT) scan revealed a well-defined and circumscribed hypodense lesion measuring 13 × 8.6 mm with multiple hyperdense foci, surrounded by endosteal sclerosis, involving the articular eminence of the TMJ, and extending posteriorly to the mandibular fossa (Fig. 2). A radiologist’s report of a magnetic resonance imaging (MRI) scan showed a surrounding bone marrow inflammatory process and anterior dislocation of the TMJ disc. The patient was operated on under general anesthesia, and surgical access to expose the TMJ was achieved via pre-auricular incision with temporal extension according to Al-Khayat and Bramely’s approach [9]. The capsule was incised, followed by retraction of the disc inferiorly after cutting through fibrous adhesions. The lesion was exposed and thoroughly curetted. Peripheral ostectomy of the cavity was performed and the TMJ capsule was sutured before closing the flap.

**Fig. 3:**
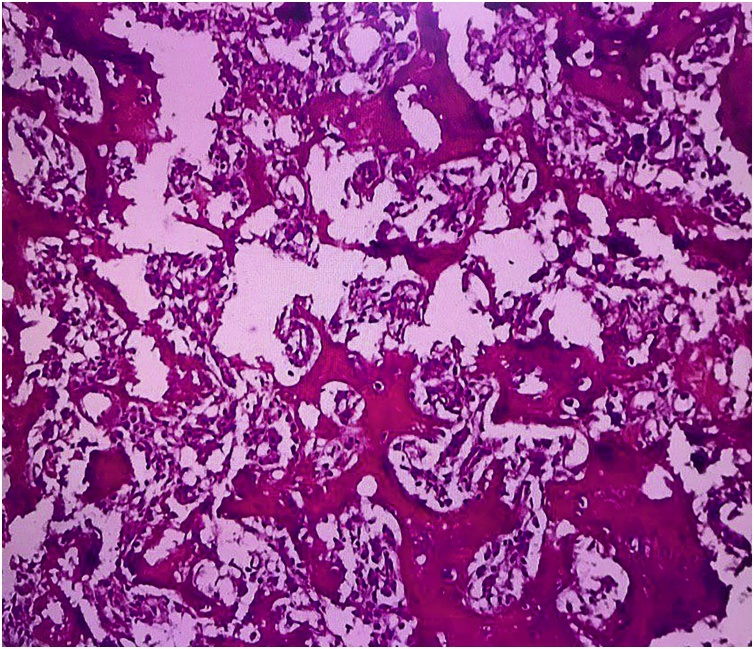
Micrograph shows irregular trabeculae of osteoid and woven bone lined by a rim of activated osteoblasts within a vascular fibrous stroma. (Hematoxylin and Eosin, 40×).

**Fig. 4:**
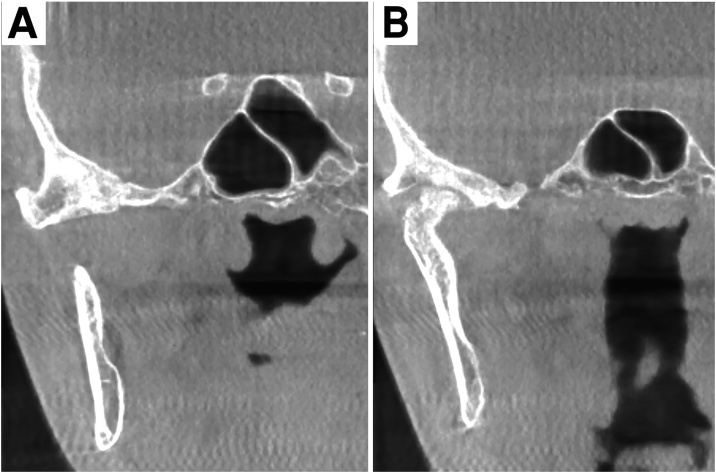
Follow up CBCT one year postoperatively. (A) Relative hypodensity of the previous surgical site in the center of endosteal sclerosis involving the surrounding bones of the skull base. (B) The residual surgical defect in glenoid fossa.

Histological examination demonstrated anastomosing trabeculae of osteoid and woven bone, lined by a rim of benign plumped osteoblasts in a loose fibrovascular stroma, and numerous multi-nucleated giant cells embedded in vascularized, fibrous connective tissue stroma (Fig. 3). One week after surgery, the patient had complete pain relief and was able to open her mouth normally. At her most recent follow-up (March 2019), 24 months after the surgical excision, the patient reported inconsistent symptoms of occasional mild pain at the surgical site elicited by lateral jaw excursion, but this did not require the administration of analgesics. No significant or relevant findings were found upon examination, except for a slight tenderness when exerting pressure over the TMJ region during jaw movement; no clicking sounds or deviation of the mandible were noted. Imaging radiography using cone beam computed tomography (CBCT) illustrated a relatively hypodense zone at the site of the previous surgery, in the center of endosteal sclerosis involving the surrounding bones of the skull base and the head of the condyle. There was also evidence of sclerotic trabeculae having formed at the site of the nidus, along with a caudal cortical thickening (Fig. 4).

## 3 Discussion

Osteoid osteoma is the third most-common benign bone tumor [7]. Although it is usually localized in the long bones and vertebrae, approximately 10%–13% of cases of osteoid osteoma develop within the joints, manifesting as intra-articular osteoid osteoma; of these cases the hip is the most commonly affected site, with the ankle, elbow, wrist, and knee being occasionally affected [3,4,7]. Only a few cases have been reported in the craniofacial region, with those reported affecting the TMJ and the jaws, showing a marked predilection for the mandible [5,6].

We conducted a search of the English-language literature held in the PubMed and ScienceDirect databases using the main search term “osteoid osteoma” and the additional search terms “maxillofacial”, “mandible”, “mandibular”, “TMJ”, “articular eminence”, “glenoid fossa”, “condyle”, “condylar”, and “jaw”. Following a detailed review of the literature, 27 reports of 29 cases of osteoid osteoma involving both the jaw and the TMJ were found and selected for this review [5,6,8,[10], [11], [12], [13], [14], [15], [16], [17], [18], [19], [20], [21], [22], [23], [24], [25], [26], [27], [28], [29], [30], [31], [32], [33], [34]], and are summarized in Table 1, along with the present case.

**Table 1:** Overview of the literature: osteoid osteomas involving the jaws and TMJ.

Case	Author (year)	Age	Gender	Site
1	Rushton 1951 [10]	27	M	Left mandibular body
2	Foss et al. 1955 [11]	26	F	Right mandibular body
3	Nelson 1955 [12]	17	M	Right posterior maxilla
4	Stoopack 1958 [13]	25	M	Left mandibular body
5	Lind and Hillerström 1964 [14]	48	M	Right condyle
6	Hillman and Brick 1965 [15]	4	F	Left antrum of maxilla
7	Greene et al. 1968 [16]	45	F	Right posterior maxilla
8	Brynolf 1969 [17]	77	M	Left anterior maxilla
9	Gupta et al. 1986 [18]	18	F	Left mandibular body
10	Zulian et al. 1987 [19]	17	F	Right mandibular ramus
11	Tochihara et al. 2001 [20]	21	F	Left condyle
12	Yang and Qiu 2001 [8]	24	F	Left articular eminence
13	Ida et al. 2002 [21]	26	F	Left mandibular body
14	Liu et al. 2002 [22]	18	M	Right anterior mandible
15	Badauy et al. 2007 [23]	26	M	Left mandibular body
16	do Egito Vasconcelos et al. 2007 [24]	23	F	Right condyle
17	Manjunatha and Nagarajappa 2009 [25]	18	F	Right mandibular angle
18	Rahsepar et al. 2009 [26]	21	M	Right subconyle
19	Karandikar 2011 [27]	14	M	Left mandibular angle
20	Singh and Solomon 2012 [28]	20	M	Left mandibular body
21	Mohammed et al. 2013 [29]	20	NS	Left mandibular body
22	An et al. 2013 [6]	10	M	Right mandibular body
23	Adouly et al 2015 [30]	11	F	Right and Left mandibular angle
24	Infante-Cossio et al. 2016 [31]	44	F	Right mandibular angle
25	Gadre et al. 2016 [32]	30	M	Left mandibular body
26	Betz et al. 2017 [33]	18	M	Right mandibular body
27	Deferm et al 2017 [5]	56	F	Right articular eminence
28	Our case	46	F	Right articular eminence

F, female; M, male; NR, not reported; TMJ, temporomandibular joint.

The criteria for selection included initially the authors' designation of bone tumors involving the jawbones or the articular surfaces of the TMJ, that histologically consistent with osteoid osteoma, the typical feature of small sized painful lesions, and a radiographic picture of a nidus containing variable degrees of opacities that surrounded mostly by bony sclerosis.

We found four cases (13.8%) of osteoid osteoma involving the maxilla; twenty-two cases (75.9%) which occurred in the mandible (eleven mandibular bodies, five mandibular condyles, one in the ramus, four mandibular angles, and one in the anterior region); and only three cases (10.3%) that involved the articular eminence of the TMJ. The patients’ ages ranged from 4 to 77 years, and the majority of lesions occurred during patients’ second or third decades of life.

The pathogenesis of osteoid osteoma remains controversial. Jaffe originally identified osteoid osteoma as a benign neoplasm [1]. Some authors have suggested that the lesion is inflammatory in origin and arises as a result of unusual reparative and healing processes [5]. In the case described here, the patient reported a history of traumatic incidents with a hard object several months prior to the onset of her symptoms, so it is possible that this history was a contributory factor in the pathogenesis she experienced.

Typically, osteoid osteoma is characterized by severe pain that gets worse at night and can be relieved by the use of NSAIDs [4].

Some researchers have demonstrated high levels of inflammatory mediators in the nidus of these lesions, specifically prostaglandin E2 and prostacyclin, elucidating the characteristic nociception and rationale for the use of NSAIDs [34,35]. However, in the case presented here, these medications were partially beneficial in relieving the pain, only at the beginning of the symptoms, with greatly negligible effect upon progression of the condition. Several studies have demonstrated that osteoid osteoma related to the joints are found to be less responsive to NSAIDs treatment than the extra-articular lesions [3,4,7,20]. That could raise the question whether these lesions contain different levels of the inflammatory mediators depending on their site of occurrence.

Generally, the intra-articular lesion is manifested as a diffuse joint pain that typically associated with a swelling and tenderness of the involved area, symptoms that might be falsely attributed to a more common intra-articular derangement, resulting in a possible delayed of diagnosis [5,7]. In accordance with that, the patient described here suffered from the symptoms for more than 2 years, and was initially misdiagnosed with TMJ internal derangement, and erroneously treated with occlusal splints and arthrocentesis procedure.

The typical radiographic feature of osteoid osteoma is that of a nidus, with a variable degree of internal calcification and surrounding sclerosis [3]. In the present case, a CT scan demonstrated these typical radiographic features, while MRI imaging demonstrated TMJ disc displacement and ongoing inflammatory processes.

Histopathological findings showed that the nidus of the lesion consists of anastomosing trabeculae of woven bone with various degrees of mineralization, uniformly scattered within a loose vascular connective tissue, rimed by prominent osteoblasts, and often accompanied by numerous osteoclast-like giant cells. Those findings are consistent with the reported detailed histopathological studies of osteoid osteoma, which further demonstrated the prominent demarcation between the nidus and the surrounding extensive sclerosis of medullary bones [36].

In differential diagnosis, considerations exist in differentiating between closely related benign osteoblastic tumours, osteoid osteoma and osteoblastoma. In the current WHO classification of bone and soft tissue tumors, osteoid osteoma and osteoblastoma are recognized as separate entities that share common features [37]. Both size and the anatomical sites of both tumors promote the notion of that separation. The designation of osteoid osteoma is principally based on the limited growth potential, with the nidus of bone lysis ranges from 1.5 to 2 cm in greatest dimension, involving mainly the cortical bone of lower extremities [4]. Whereas osteoblastomas demonstrate a larger size >2 cm, and up to 10 cm in aggressive patterns, with the medullary bone of axial skeleton are affected in majority of cases [36]. In a novel study, Barlow et al proposed reclassification of both osteoid osteoma and osteoblastoma as a single entity, demonstrating that both share identical novel histopathological and immunohistochemical features, which should negate the current concept of separation [38]. However, the search in English literature is still governed by the long-established concept of separating these osteoblastic tumors, in both nomenclature and description, which could form a bias in reviewing either of the two phenotypes separately.

Surgical intervention remains the most commonly recommended treatment option for osteoid osteoma [6,8,19,22]. Recently, however, minimally invasive interventions such as CT- or MRI-guided radiofrequency ablation (RFA) and CT-guided laser photocoagulation have been developed [39].

## 4 Conclusion

Intra-articular Osteoid osteoma is a rare site of occurance, and usually exhibits nonspecific symptoms compared to those in other locations, leading to misdiagnosis and delayed treatment. In the TMJ, in the majority of reported cases, the lesion involved the condylar head among other articular surfaces. In this article, we have reported a case of intra-articular osteoid osteoma involving the articular eminence and glenoid fossa of the TMJ, which, to our knowledge, has been only reported few times in the literature. CT images were helpful in displaying the typical radiographic features and localization of the lesion. Surgical intervention was performed to excise the lesion, which resulted in the complete relief of symptoms and uneventful healing of the area. Histological examination confirmed the diagnosis of osteoid osteoma.

## Sources of funding

All authors declare that there is no any sources of funding.

## Ethical approval

Ethnical approval is exempt from my institution.

## Consent

Written informed consent was obtained from the patient for publication of this case report and accompanying images. A copy of the written consent is available for review by the Editor-in-Chief of this journal on request.

## Author contribution

HAA: Conceptualizing and writing of the paper.

SAI and HAA: Assistant to writing of the manuscript and involved in care of patient.

ASAA: Editing of manuscript.

SAI and HAA: Main surgeon involved in care of patient and final editor of manuscript.

## Registration of research studies

NA.

## Guarantor

Ahmed Alshewered

## Provenance and peer review

Not commissioned, externally peer-reviewed.

## Declaration of Competing Interest

All authors declare that there is no any conflicts of interest.
